# Resuscitation Resident Impact in the Treatment of Sepsis

**DOI:** 10.7759/cureus.9257

**Published:** 2020-07-18

**Authors:** Michael J Burla, Nashid Shinthia, Judith A Boura, Lihua QU, David A Berger

**Affiliations:** 1 Emergency Medicine, Beaumont Health System, Royal Oak, USA; 2 Emergency Medicine, Southern Maine Health Care, Biddeford, USA; 3 Emergency Medicine, Baylor University Medical Center, Houston, USA; 4 Research, Ascension Macomb-Oakland, Warren, USA; 5 Research, Beaumont Health System, Royal Oak, USA

**Keywords:** resuscitation, emergency department, sepsis, septic shock, residency

## Abstract

Background

The resuscitation of septic patients is a fundamental skill of emergency medicine (EM) training. We developed a required rotation designed to augment resident training in resuscitating critically ill patients in the emergency department (ED). The purpose of this study was to evaluate the successful completion of sepsis core measures alongside clinical outcomes between patients with a resuscitation resident (RR) involved in care versus patients without.

Methods

This retrospective study was conducted at a single site tertiary care Level 1 trauma center with an ED census of 130,000 visits annually. Data were collected from January 1, 2015, to December 31, 2016, using the electronic medical record (EMR) via an Epic query (Epic Systems Corp., Verona, WI). Patients admitted with severe sepsis or septic shock (Surviving Sepsis Campaign guidelines) were included and separated into two groups, one with RR involvement and one without. Emergency department length of stay, time to initial lactic acid draw, lactic acid value, time to bolus fluid initiation, time to antibiotic initiation, need for medical intensive care unit (ICU) admission, and 30-day mortality were compared between the two groups. Chi-square tests and Fisher’s exact tests were used to analyze the categorical variables. Two-sided t-tests and Wilcoxon rank-sum tests were used to examine continuous variables.

Results

Out of 4,746 patients admitted, 101 patients had an RR participate in their care. The median time to initial lactic acid draw was shorter (0.53 vs 1.05 hours; p < 0. 0001) and the lactic acid level was higher (2.5 vs 1.8 mmol/L; p < 0. 0001) with the presence of an RR. Resuscitation resident was correlated with a decrease in time to antibiotics and appropriate 30 cc/kg bolus, however, these were not statistically significant (p = 0.10 and p = 0.09 respectively). Resuscitation resident involvement was also associated with more medical ICU (45.5% vs 18.8%; p<0.0001) admissions and a higher 30-day mortality (14.9% vs 29.7%; p < 0. 0001). All other variables were not statistically significant.

Conclusion

Resuscitation residents demonstrate a statistically significant impact on lactic acid-related bundle compliance and help facilitate the care of higher acuity severe sepsis and septic shock patients.

## Introduction

The resuscitation of severe sepsis and septic shock patients in the emergency department (ED) is an integral aspect of emergency medicine. Extensive strides have been made to improve outcomes in this patient population, beginning with the landmark description of early goal-directed therapy in 2004 [1], to the subsequent studies of the Protocolized Care for Early Septic Shock (ProCESS) and Australasian Resuscitation in Sepsis Evaluation (ARISE) trials [2-4]. These subsequent trials demonstrated that protocol-based resuscitation did not improve patient outcomes. Given the high morbidity and mortality of sepsis and septic shock, the Surviving Sepsis Campaign was initiated globally to help improve the treatment of sepsis and septic shock [5]. The campaign involves a six-prong approach, with sepsis core measures being one of the prongs [6]. The initial core measures include obtaining blood cultures, lactic acid, starting board-spectrum antibiotics, and starting a 30 cc/kg fluid bolus when necessary, within the first three hours of a patient’s presentation.

Achieving the initial sepsis core-bundle within three hours can be challenging, and sepsis may not always be obvious based on the initial presentation. For this reason, a variety of approaches have been developed to maximize adherence to the sepsis core measures [7-11]. One method that could conceivably improve sepsis bundle achievement is having additional staff specifically designed to help with the resuscitation of patients that are critically ill. At our institution, we have developed a novel one-month rotation dedicated to resuscitative care in the ED [12]. The purpose of this curriculum was to help supplement our residents' resuscitation education.

While this curriculum was developed for educational purposes, we hypothesize that having a resuscitation resident involved will improve patient care. Having an additional resident participating in the care of acutely ill populations, including patients with severe sepsis and septic shock, could lead to interventions and treatment plans initiated more efficiently. Given this potential, we performed a retrospective study assessing resuscitation resident impact on patient outcomes and the achievement of sepsis core measures on patients with severe sepsis and septic shock in the ED.

## Materials and methods

Overview

This was a retrospective study, conducted at a single-center tertiary hospital, with an ED census of ~130,000 annually. Our institution’s admission rates have been approximately 30% overall and 17% in the intensive care unit (ICU).

Course description

As a required rotation within our standard post-graduate year 2 (PGY-2) curriculum, the resuscitation rotation is a one-month experience focused on the assessment and management of high-acuity patients. The location of this course is at our primary campus, a large level 1 trauma center, with a medical school affiliation. One to two residents are designated per month and are expected to be available in the department for a minimum of 40 hours a week, mostly during the highest volume hours (10 am - 6 pm). The target population that is focused on is the acutely ill, which includes patients with severe sepsis and septic shock. During this rotation, the resuscitation resident (RR) is expected to help facilitate the care of these patients, as well as augment resuscitative efforts. These residents do not take over as the primary caregiver but rather aid with procedures, monitoring, speaking with consultants, and disposition. The RR identifies which patients to become involved with via the guidance of the attending on shift, by screening patients in the high-acuity area of the department. The curriculum includes weekly didactics, required reading, article discussions, and pre/post-exam.

Design

Data collection was approved by our institution’s Institutional Review Board (IRB). Sepsis encounters were identified in the ED over a two-year time frame (January 2015 - December 2016) via a query of our facility’s electronic medical record (EMR, Epic by Epic Systems Corp., Verona, WI). These encounters were discovered utilizing sepsis codes from the International Classification of Diseases, 9th (995.91 - 995.92) and 10th (A41 - A41.9) revisions, Clinical Modification (ICD 9/10-CM). Resident encounters were independently recorded in a protected, web-based data bank, with individual login (New Innovations by New Innovations Inc., Uniontown, OH). These encounters were then compared to encounters of sepsis patients without RR involvement. The medical record numbers (MRN) of the patients were utilized to compare encounters. All Epic data were exacted by an experienced data user (LQ), and relevant clinical information was organized into a database. All data were managed on a password-protected platform (SharePoint by Microsoft Corp., Redmond, WA), with access granted only to key personnel.

Study population criteria

**Figure 1:**
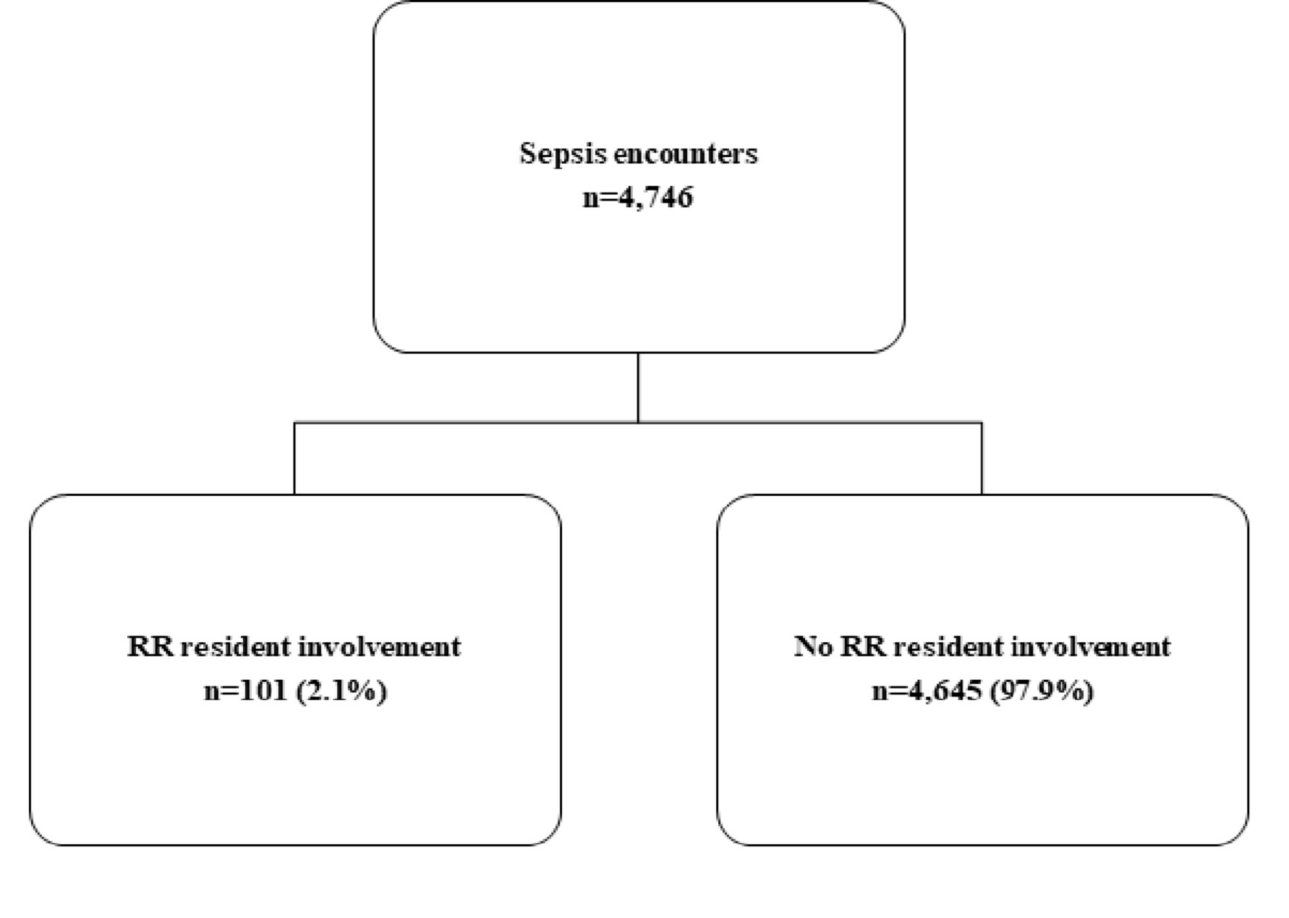
Flow diagram of sepsis encounters

A total of 4,746 encounters were observed. Eligible encounters were included in enrollment if they were ≥ 18 years of age, met sepsis coding, and were admitted to the hospital. Pediatric and pregnant patients were excluded. As stated in the study design, patients were separated into two groups, one with RR involvement, and one without (Figure 1).

The basic demographics of age, sex, and body mass index (BMI) were compared between groups, as well as sepsis bundle adherence. Sepsis bundle parameters included time to initial lactic acid, second lactic acid order and time to order in the appropriate setting, time to antibiotics, and 30 cc/kg bolus and time to bolus as indicated. Lactic acid values, ICU admission, ED length of stay, and 30-day mortality were also compared between groups.

Outcome measures

The primary outcome of this study was to compare 30-day mortality between the patients who received RR care and those who did not. Secondary outcomes were to compare if sepsis bundle parameters were met between each group.

Data analysis

The analysis was completed by an experienced biostatistician (JB). Missing data remained missing and were not replaced with substitutions or interpolation. We compared several variables between patients who had RR involvement versus those that did not. Categorical variables were examined using Pearson’s Chi-square where appropriate (expected frequency >5); otherwise, Fisher’s exact tests were used. Age was examined using a two-sample t-test. The remaining continuous variables were examined using Wilcoxon rank tests. A p-value of <0.05 was considered statistically significant. SAS for Windows® version 9.3 (SAS Institute, Cary, NC) was used for all analyses.

## Results

The demographics of patients and sepsis bundle variables who had RR involvement as compared to those that did not are illustrated in Table 1.

**Table 1:** Demographics of patients with RR involvement and without RR: resuscitation resident; BMI: body mass index; ICU: intensive care unit; ED: emergency department; LOS: length of stay

	RR involved N=101	No-RR involved N=4,645	P-Value
Age mean+/-SD (median) Min to max	67.6+/-16 (68) 18 to 94	65.5+/-19 (68) 18 to 106	0.27
BMI mean+/-SD (median) Min to max	N=101 29.9+/-7.9 (28.6) 16.6 to 52.1	N=4,225 28.9+/-8.5 (27.3) 11.0 to 105.7	0.14
Males	50 (49.5%)	2,333 (50.2%)	0.89
Time to lactic acid (hrs) Median (25^th^, 75^th^) Min to max	N=91 0.53 (0.20, 1.63) 0.03 to 1016	N=3,465 1.05 (0.45, 4.38) -21.3 to 1418	<0.0001
Lactic acid values Median (25^th^, 75^th^) Min to max	N=91 2.5 (1.5, 4.6) 0.5 to 22.1	N=3,465 1.8 (1.2, 2.7) 0.4 to 22.9	<0.0001
Time to 2^nd^ lactic acid-hrs Median (25^th^, 75^th^) Min to max	N=67 7.7 (4.6, 19.2) 1.4 to 1025	N=1,883 16.0 (7.8, 52.6) 1.3 to 1501	<0.0001
Time to 2^nd^ lactic acid-hrs ≤ 3 hours	1/67 (1.5%)	37/1,883 (2.0%)	1.00
Time to 2^nd^ lactic acid-hrs ≤ 6 hours	27/67 (40.3%)	284/1,883 (15.1%)	<0.0001
2^nd^ lactic acid values Median (25^th^, 75^th^) Min to max	N=67 2.5 (1.6, 5.5) 0.7 to 12.3	N=1,884 1.8 (1.2, 2.9) 0.1 to 19.2	0.0002
Time to antibiotics (hrs) Median (25^th^, 75^th^) Min to max	N=96 4.26 (2.58, 6.65) 0.70 to 373	N=4,396 4.60 (3.02, 8.93) 0.07 to 1042	0.10
Time to bolus (hrs) Median (25^th^, 75^th^) Min to max	N=87 1.67 (0.85, 5.15) 0.17 to 310.3	N=3,601 1.93 (1.10, 3.77) 0.08 to 1279	0.18
30cc/kg bolus within 3 hours	9/60 (15.0%)	211/2,429 (8.7%)	0.09
ICU admission	46 (45.5%)	871 (18.8%)	<0.0001
ED LOS hr Median (25^th^, 75^th^) Min to max	6.9 (5.0, 8.3) 0.7 to 27.0	6.8 (5.0, 9.6) 0.02 to 48.5	0.45
30-day mortality	30 (29.7%)	690 (14.9%)	<0.0001
Days to death Median (25^th^, 75^th^) Min to max	N=30 8.2 (2.5, 17.5) 0 to 20.5	N=690 9.0 (3.6, 16.3) 0 to 29.9	0.40

A total of 4,746 patient encounters were observed during the time period. From these encounters, 101 (2.1%) of the patients had an RR involved. There was no evidence of any statistically significant differences between group baseline demographics, although comorbidities were not accounted for. Patients who had RR involvement did have a shorter time to initial lactic acid (0.53 hours vs 1.03 hours; p < 0.0001) and second lactic acid (7.7 hours vs 16.0 hours; p < 0.0001). In addition, RR involvement was associated with a higher percentage of patients achieving time to second lactic acid under the six-hour mark as compared to non-RR involvement (40.3% vs 15.1%; p < 0.0001). Encounters with RR involvement also experienced decreased time to antibiotics and more patients received 30 cc/kg fluid boluses when appropriate; however, these results were not statistically significant (p = 0.10 and p = 0.09, respectively).

Regarding the severity of illness, RR involvement was associated with higher lactic acid values, both with the first lactic acid (p < 0.0001) and second lactic acid (p = 0.0002), as well as higher ICU admission rates (45.5% vs 18.8%; p < 0.0001) and 30-day mortality (29.7% vs 14.9%; p <0.0001). For this reason, further analysis was done to assess the subset of ICU patients in both groups, as illustrated in Table 2.

**Table 2:** Demographics of ICU patients with RR involvement and without ICU: intensive care unit; RR: resuscitation resident; ED: emergency department; LOS: length of stay; BMI: body mass index

	RR involved N=46	No RR involved N=871	P-Value
Age mean+/-SD (median) Min to max	67+/-15 (66) 33 to 91	67+/-17 (68) 18 to 106	0.96
BMI mean+/-SD (median) Min to max	30.6+/-8.3 (29.3) 16.6 to 52.1	29.4+/-8.8 (27.7) 11.0 to 105.7	0.30
Males	18 (39.1%)	467 (53.6%)	0.055
Time to lactic acid (hrs) Median (25^th^, 75^th^) Min to max	N=41 0.4 (0.2, 2.0) 0.03 to 1016	N=823 1.0 (0.3, 7.5) -15 to 1418	0.008
Lactic acid values Median (25^th^, 75^th^) Min to max	N=41 3.9 (1.7, 6.8) 0.8 to 22.1	N=823 2.2 (1.4, 3.5) 0.5 to 22.1	0.002
Time to 2^nd^ lactic acid-hrs Median (25^th^, 75^th^) Min to max	N=37 6.1 (4.5, 14.5) 1.4 to 1025	N=699 13.4 (7.1, 65) 1.7 to 981	0.0002
Time to 2^nd^ lactic acid-hrs ≤ 3 hours	1/37 (2.7%)	17/699 (2.4%)	0.61
Time to 2^nd^ lactic acid-hrs ≤ 6 hours	18/37 (48.7%)	111/699 (15.9%)	<0.0001
2^nd^ lactic acid values Median (25^th^, 75^th^) Min to max	N=37 3.1 (1.6, 5.9) 0.7 to 12.3	N=700 1.9 (1.3, 3.3) 0.1 to 19.2	0.006
Time to antibiotics (hrs) median (25^th^, 75^th^) Min to max	N=44 4.7 (2.5, 7.8) 0.7 to 373	N=835 4.9 (2.7, 15.4) 0.5 to 1042	0.34
Time to bolus (hrs) Median (25^th^, 75^th^) Min to max	N=42 1.3 (0.8, 5.4) 0.3 to 310	N=782 1.9 (0.9, 5.8) 0.1 to 661	0.35
30cc/kg bolus within 3 hours	7/29 (24.1%)	75/475 (15.8%)	0.30
ED LOS hr Median (25^th^, 75^th^) Min to max	7.1 (5.5, 9.1) 0.7 to 27.0	6.8 (5.0, 9.7) 0.4 to 36.2	0.58
30-day mortality	15 (32.6%)	301 (34.6%)	0.79
Days to death Median (25^th^, 75^th^) Min to max	N=15 9.4 (1.4, 17.9) 0 to 20	N=301 10.6 (4.3, 16.9) 0 to 30	0.51

This analysis of ICU only patients demonstrated no difference in basic demographics or 30-day mortality between patients with RR involvement and ones without. In addition, RR involvement was still associated with statistically significant shorter median time to first and second lactic acid (p = 0.008; p = 0.0002), as well as statistically significant higher lactic acid values (p = 0.002; p = 0.006). Lastly, RR involvement was associated with the second lactic acid being acquired ≤ 6 hours (p < 0.0001).

## Discussion

Overall, our investigation demonstrated that RR involvement was associated with statistically significant improvement in the fulfillment of two aspects of sepsis bundle compliance. The first aspect was reduced time to initial lactic acid draw, and the second was achieving the second lactic acid draw under the six-hour mark. With respect to clinical outcomes and 30-day mortality, the initial analysis revealed that the RR arm did have a higher rate of 30-day mortality, though this effect was not observed when only including ICU admissions in each cohort. We suspect that the change in mortality is due to making the cohort's severity of illness more comparable, as the RR is typically only involved in the care of critically ill patients. In addition, patients who had RR involvement had a quicker time to appropriate fluid bolus and antibiotics, however, these findings were not statistically significant (p = 0.09 and p = 0.10, respectively). Given the small percentage of overall RR encounters, we suspect that more RR involvement could have potentially led to these findings being statistically significant.

As stated in the introduction, the resuscitation rotation is a novel curriculum designed to augment resuscitative training [12]. Since this is a novel curriculum, there is no specific current literature that we have seen to compare our findings. However, there have been several studies assessing ways to achieve sepsis core measures and attempt to improve patient outcomes [13]. Most of these studies focus on a sepsis alert protocol, either involving personnel or electronically driven. One model that has been studied is the implementation of a best practice alert (BPA) in the EMR to alert clinicians of possible sepsis patients. Studies have demonstrated a BPA leading to decreased time to antibiotics and other parameters [14-15], nevertheless, it did not lead to an improvement in mortality [14-15]. Personnel driven models, either with a sepsis response team or triage protocol, have also had varying degrees of success with decreasing time to sepsis bundle parameters [7-11], with only one demonstrating a possible mortality benefit [16]. Overall, similar to our study, the vast majority of these interventions have led to some improvement in achieving different sepsis core measurements [7-11,13-17]. The difference in this approach is that the resuscitation rotation was designed to be educational in nature, where the above-mentioned interventions were all specifically designed to improve outcomes for sepsis patients. Other educational programs have been developed in the past to improve the proficiency of resuscitative training [18-19], but none appear to measure patient outcomes.

While this study is specific to assessing sepsis patient outcomes with RR involvement, emergency medicine physicians in our department were anecdotally noticing expedited care when the RR was working in general. For this reason, faculty at our institution decided that it might be of interest to evaluate patient-centered outcomes with RR involvement. This way, data can be collected not only to improve the residents' educational experience but also to assess how patient care can be improved. While the number of RR cases in this particular study are limited, we are continuing to develop ways to get RR involvement in patient care. In addition to examining the impact of a RR on septic patients, we are currently investigating the impact on other types of critical patients to include those suffering from cardiac arrest.

Improving resuscitative education and retention of training has proven to be a difficult task [20]. The American Heart Association's recent scientific statement recognizes the concern of decay in resuscitative knowledge and skills over time [21]. Moreover, it has also been suggested by the American Association of Medical Colleges that a gap exists between educational objectives and successful resuscitation practices in graduate medical education [20]. These concerns are likely multifocal, however, one contributing factor could be the variation in resident experience [22]. Since real-time resuscitations can be rare compared to other clinical scenarios, some residency programs have incorporated simulation or specific didactics to supplement resuscitative training. Simulation has been a great tool to provide educational opportunities for rare clinical scenarios, however, actual effectiveness may vary [23]. Our rotation offers a combination of hands-on experience and didactics to standardize the resident experience as much as possible. The rotations didactics are weekly, focusing on sepsis resuscitation and other resuscitative education. In addition, there is a pre- and post-test to the rotation, implemented to discover and target the weaknesses of each individual resident. As stated in the introduction, dedicated rotations have been shown to improve competency in a given subject [24]. Residents that have completed the rotation have reported an increase in resuscitative confidence, as well as a decrease in anxiety while preforming some resuscitative procedures [12]. We believe that the resuscitation rotation curriculum has benefited our residents and provides a dedicated month of hands-on resuscitation experience that has the potential to improve patient care as well.

This was a retrospective, single-center study, with several limitations. First, it is hard to control for all confounding variables with retrospective data. Secondly, encounters, where RR was involved, were logged at the discretion of the resident. For this reason, it is possible some encounters had RR involved that were not logged by error. In addition, there was a significant imbalance between cohorts, where the majority of sepsis encounters did not have an RR involved. The small percentage of RR involvement could have led to a deficiency in data, resulting in time to fluid bolus and antibiotics not being statistically significant. We believe that this discrepancy is due to patients who met the ICD9/10 coding threshold for severe sepsis and septic shock, however, presented clinically as low acuity, for which the RR would not be involved in care. This is also supported by the fact that RR involvement was associated with higher initial lactic acid levels and higher mortality rates. It is also likely that many cases arrived outside the RR scheduled work hours. For this reason, a secondary analysis was done of ICU admission patients, which demonstrated no statistical difference in the mortality rate. It is difficult to speculate why RR involvement did not lead to a mortality benefit when controlling for ICU patients; however, there are a variety of factors and confounding variables during a patient's hospitalization that could not be accounted for. Finally, some data points were missing from the retrospective collection, which were not replaced in the analysis, thus potentially altering our findings.

## Conclusions

Septic patients with RR involvement demonstrated a statistically significant benefit to initial and repeat lactic acid draws. While other sepsis bundle criteria did not meet statistical significance, times to appropriate fluid bolus and antibiotics were shorter when a RR was involved in care. This curriculum was designed to augment residents’ resuscitation education; however, it does have the potential to improve patient care of the critically ill, including septic patients.
